# Walking recovery after tethered cord release

**DOI:** 10.1007/s10143-024-02497-8

**Published:** 2024-06-25

**Authors:** Mohamed AR AbdelFatah, Aly Ibrahim, Sameh Hefny

**Affiliations:** Neurosurgery Department, Faculty of Medicine, Ain Shams University, Cairo, Egypt

**Keywords:** Myelomeningocele, Spinal cord release, Tethered cord, Untethering, Walking recovery

## Abstract

After myelomeningocele (MMC) repair, a secondary tethered spinal cord occurs in almost all patients. The tethered spinal cord may result in progressive neurological deterioration and walking disability. This retrospective cohort study aimed to highlight the walking recovery one year after tethered cord release and its relation to the preoperative conus level. We reviewed the medical records at our university hospital from January 2014 to December 2022. The patients who underwent spinal cord untethering following lumbosacral MMC repair were included. We assessed the walking recovery one year after cord release using the modified Benzel scale. Thirty-seven patients met our selection criteria. There were 19 girls (51.4%) and 18 boys (48.6%). Their mean age at presentation was 8.6 years. The preoperative conus vertebral levels ranged between L4 and S3. One year after spinal cord release, 37.8% of the patients regained their walking ability. All the patients whose preoperative conus level was at S2 or S3 regained their walking ability. In contrast, all the patients with preoperative conus levels at L4 or L5 didn’t regain their ability to walk. One-third (33.3%) of patients whose conus was at the S1 level regained their walking ability one year after cord release. One year after tethered cord release, 37.8% of the patients regained their walking ability. We found that the walking recovery was statistically associated with the preoperative conus level. A multicenter prospective study is required to support the results of this study.

## Introduction

Myelomeningocele (MMC) is an open neural tube defect that results from incomplete intrauterine closure of the neural tube [1].

Secondary tethered spinal cord (TSC) occurs in nearly all patients following MMC repair. The tethered, low-lying conus medullaris results from adhesions between the spinal cord, nerve roots, and the repaired dura [2].

The tethered cord may result in progressive neurological deterioration and walking disability [3].

Spinal cord release (untethering) from the tethering tissues is believed to improve neurological deficits [4, 5].

This study investigated the walking recovery one year after cord untethering and its relationship to the preoperative conus level.

## Materials and methods

This is a retrospective, single-arm cohort study. We reported this cohort study following the STROBE “Strengthening the Reporting of Observational Studies in Epidemiology” principles.

We carried out the study under the Code of Ethics of the World Medical Association (Declaration of Helsinki) for studies involving humans.

The study protocol was approved by the research ethics committee of the faculty of medicine at our university (FWA 000017585). We obtained informed consent as required to use the collected data of the patients anonymously.

We reviewed the medical records at our university hospitals for the data of the patients with the following criteria:

*Eligibility criteria*

- Patients who underwent lumbosacral MMC repair in their first week of life.
- Patients who developed deterioration in their walking ability before cord untethering.
- Patients who underwent spinal cord untethering procedures in our university hospital from January 2014 to December 2022.

*Exclusion criterion*

- Recurrent cases after untethering.

The collected data included patients’ demographics, preoperative neurologic state, preoperative and postoperative conus levels, and walking ability one year after spinal cord release.

The walking ability was assessed using the modified Benzel scale, as illustrated in Table 1 [6]. Grade V is the first grade where ambulation is observed and represents the ability to walk at least 25 feet with or without assistance.

**Table 1:** Modified Benzel scale

Modified Benzel Grade	Description
**I**	No motor or sensory function is preserved in sacral segments S4-5.
**II**	Sensory but no motor function is preserved in sacral segments S4-5.
**III**	Unable to walk; the majority of the key muscles of the lower limbs have a muscle grade > 3.
**IV**	Unable to walk; some functional motor control is significantly useful (e.g., assist in transfers) but insufficient for independent walking.
**V**	Limited walking; motor function allows walking with or without assistance, but significant problems secondary to lack of endurance or fear of falling limit patient mobility (must be able to ambulate at least 25 feet).
**VI**	Unlimited walking; ambulatory without assistance and significant limitations other than slightly uncoordinated gait (must be able to ambulate at least 150 feet without a helper).
**VII**	Neurologically intact except for minimal deficits that cause no functional difficulties (must have a neurologically normal gait and be able to walk without assistive devices)

Walking recovery is defined as the ability to walk at least 25 feet with or without assistance, i.e., modified Benzel scale grades V, VI, or VII.

Preoperative magnetic resonance imaging (MRI) of the whole spine was done for all patients. Pediatric neurosurgeons performed the operations.

A perioperative prophylactic third-generation cephalosporin was administered to all patients. Antibiotics were administered 30 min before the skin incision and continued for at least three days.

All the patients underwent microscopic cord and nerve root release of all the tethering elements under electrophysiological monitoring, followed by dural repair.

The patients were discharged with instructions to attend two to three physiotherapy sessions per week. The patients were followed up after the untethering procedure for a mean period of 2.4 years (ranging from 1 to 6 years).

An MRI of the spine was performed three months and one year after surgery to assess the level of the conus medullaris.

We conducted the statistical analyses using SPSS version 21 (IBM Corp., Armonk, New York, USA). Quantitative data was described by the mean and range, while qualitative data was described by frequencies. A chi-square test of independence was used to determine the association between the preoperative conus level and the walking recovery one year after cord release.

A p-value < 0.05 was considered statistically significant.

## Results

Thirty-seven patients met the selection criteria of our study. There were 19 girls (51.4%) and 18 boys (48.6%). Their mean age at the time of the untethering procedure was 8.6 years (ranging from 5.8 to 11.6 years).

The patients underwent MMC repair in the first week of life. Thirty-one patients (83.7%) had ventriculoperitoneal shunts (VPS). The shunts were clinically functioning at the time of spinal cord release and one year later. The average age of VPS placements was 10.3 months (range: 1 to 22 months).

The mean duration of the inability to walk before cord release was 29.4 days (ranging from 22 to 37 days).

The preoperative conus vertebral levels ranged between L4 and S3. One year after spinal cord release, 14 patients (37.8%) regained walking ability.

Six patients (16.2%) suffered from postoperative wound cerebrospinal fluid (CSF) leaks. They were managed by prone positioning, secondary sutures, and repeated wound dressings, and the CSF leak stopped within a few days.

Two patients developed postoperative superficial wound infections that improved with wound debridement and intravenous antibiotics within two weeks.

One patient (2.7%) had postoperative neurological deterioration and did not improve even one year after surgery. He suffered from a secondary inability to walk for 37 days. The preoperative conus level of this patient was at L5. His preoperative modified Benzel scale was grade IV, and he immediately postoperatively became grade III.

The preoperative conus level and the walking recovery one year after cord untethering are shown in Table 2.

**Table 2:** The preoperative conus level and the walking ability one year after cord untethering using the modified Benzel scale

Preoperative conus level	N	Preoperative Modified Benzel scale		One year after cord untethering Modified Benzel scale							*P*-value
		III	IV	I	II	III	IV	V	VI	VII	
**L4**	2	0	2	0	0	0	2	0	0	0	0.0001*
**L5**	11	4	7	0	0	5	6	0	0	0	
**S1**	15	11	4	0	0	2	8	**5**	0	0	
**S2**	6	4	2	0	0	0	0	**4**	**2**	0	
**S3**	3	2	1	0	0	0	0	0	**3**	0	

N: Number of patients *Chi-Square Test of independence

The walking recovery one year after cord release was statistically significantly associated with the preoperative conus level (P-value = 0.0001).

All the patients with preoperative conus levels at L4 or L5 were unable to walk one year after cord untethering. All the patients whose preoperative conus level was at S2 or S3 regained their walking ability one year after cord release.

In addition, one-third (33.3%) of the patients whose conus was tethered at the S1 level regained their ability to walk one year after cord release.

The preoperative and postoperative conus levels of the included cases are illustrated in Table 3.

**Table 3:** The number of cases at each conus level

Conus level	Preoperative	Three months after surgery	One year after surgery
**L2**	0	0	1
**L3**	0	0	8
**L4**	2	5	13
**L5**	11	14	12
**S1**	15	13	3
**S2**	6	3	0
**S3**	3	2	0

### Illustrative case

A 10-year-old girl presented with a secondary inability to walk for 23 days. She underwent MMC repair in her first week of life. She had a clinically functioning VPS, which was placed in her eighth month of age. Her preoperative modified Benzel scale was IV, and the conus level was at S1. She underwent an uneventful cord untethering with no postoperative complications. She was able to walk 30 feet without assistance one month after the operation. Her modified Benzel scale one year after the operation was V.

## Discussion

This study is unique as it highlighted the walking recovery after tethered cord release secondary to MMC repair. One year after spinal cord release, 37.8% of the patients regained their walking ability.

We found that all the patients whose preoperative conus level was at S2 or S3 regained their walking ability one year after cord release, but none of the patients whose preoperative conus level was at L4 or L5 could walk.

One-third of the patients whose conus was tethered at the S1 level could walk one year after cord release.

The results of this study found that the walking recovery one year after cord release was statistically related to the preoperative conus level. This finding helps the patients and their families develop realistic expectations regarding their outcomes.

In this study, 62.2% of the patients did not regain their ability to walk after spinal cord release. Similarly, Sun et al. found that almost half (47.4%) of patients reported no improvement after untethering surgery. Moreover, 40.2% of their patients experienced motor deterioration [7].

Other studies reported that up to 72% of MMC patients had gait improvement following the release of the tethered cord. However, these studies did not correlate the gait improvement with the preoperative conus level [2, 8].

In our study, one patient (2.7%) had postoperative neurological deterioration and did not improve even one year after surgery. The preoperative conus level of this patient was at L5.

This study estimates the walking recovery after tethered cord release. This encourages the parents of MMC patients to seek medical advice upon any deterioration in the patient’s walking ability.

The association of the walking recovery after cord release with the preoperative conus level is not well understood. However, the better outcome with distal sacral conus may be related to the already congenitally elongated roots, which could tolerate untethering and traction.

The limitations of this study are its retrospective nature, the small number of patients, and the single-center experience. In addition, the absence of high-quality MRI images for the included patients was a drawback in this study. A multicenter prospective study will be helpful to support the results of this study.

## Conclusion

One year after the tethered cord release, 37.8% of the patients regained their walking ability. We found that the walking recovery was statistically associated with the preoperative conus level. A multicenter prospective study will be helpful to support the results of this study.

## Data Availability

No datasets were generated or analysed during the current study.
